# The posterior/medial dry needling approach of the tibialis posterior muscle is an accurate and safe procedure: a cadaveric study

**DOI:** 10.1186/s12891-022-05530-3

**Published:** 2022-06-14

**Authors:** Albert Pérez-Bellmunt, Carlos López-de-Celis, Jacobo Rodríguez-Sanz, Shane L. Koppenhaver, Daniel Zegarra-Chávez, Sara Ortiz-Miguel, César Fernández-de-las-Peñas

**Affiliations:** 1 Faculty of Medicine and Health Sciences, Universitat Internacional de Catalunya (UIC-Barcelona), C/ Josep Trueta S/N Sant Cugat del Vallès, Barcelona, Spain; 2 ACTIUM Functional Anatomy Group, Barcelona, Spain; 3 Baylor University Doctoral Program in Physical Therapy, Waco, TX USA; 4 Department of Physical Therapy, Occupational Therapy, Physical Medicine and Rehabilitation, Universidad Rey Juan Carlos (URJC), Alcorcón, Madrid, Spain; 5 Clínica E Investigación en Fisioterapia: Terapia Manual, Cátedra Institucional en Docencia, Punción Seca Y Ejercicio Terapéutico, Universidad Rey Juan Carlos, Alcorcón, Madrid, Spain; 6 Facultad de Ciencias de La Salud, Universidad Rey Juan Carlos, Avenida de Atenas s/n, 28922 Alcorcón, Madrid, Spain

**Keywords:** Tibialis posterior, Dry needling, Cadaver, Anterior approach, Medial approach, Safety

## Abstract

**Background:**

Evidence suggests that tibialis posterior muscle plays an important role in equinovarus foot deformity in patients who had suffered a stroke and it is one of the most frequently injected lower-extremity muscles for the management of spasticity. Our aim was to assess if a needle accurately and safely penetrates the tibialis posterior muscle during the application of dry needling.

**Methods:**

We conducted a cadaveric descriptive study. Needling insertion of the tibialis posterior was conducted in 11 cryopreserved cadavers with a 70 mm needle. The needle was inserted using two common approaches, at midpoint (posterior/medial approach) and at upper third (anterior approach) of the leg towards the tibialis posterior. The needle was advanced into the tibialis posterior based upon clinician judgement. Cross-sectional anatomical dissections were photographed and analyzed by photometry. Safety was assessed by calculating the distances from the tip and the path of the needle to proximate neurovascular structures.

**Results:**

Accurate needle penetration of the tibialis posterior muscle was observed in all cadavers with both approaches. In general, distances from the needle to the neurovascular bundles were larger with the posterior/medial approach than with the anterior approach, reaching statistically significance for needle tip to nerve (mean difference: 0.6 cm, 95%CI 0.35 to 0.85 cm) and vascular bundle (mean difference: 0.55 cm, 95%CI 0.3 to 0.8 cm) distances (*P* < 0.001) and needle path to vascular bundle distance (difference: 0.25 cm, 95%CI 0.1 to 0.4 cm, *P* = 0.045). Age and gender did not influence the main results.

**Conclusions:**

This cadaveric study suggests that needling of the tibialis posterior muscle can be accurately and safely conducted. Safety seems to be larger with the posterior/medial approach when compared with the anterior approach.

## Background

Dry needling is an invasive procedure with an increasing number of practitioners in clinical practice; however, the presence of potential adverse events suggests the need for research identifying the risks of dry needling [1]. Although most adverse events seem to be minor (e.g., mild bleeding, bruising, and pain during the intervention), potential major events (e.g., weakness, numbness) can also occur [2]. In fact, McManus & Cleary described a lasting and debilitating neuropraxia of the radial nerve after the application of dry needling [3].

Superficial anatomical landmarks and knowledge of deeper anatomic structures are used to apply dry needling; however, its application on deep muscles is controversial due to the possibility of needling surrounding tissues such as nerve trunks or vessels. The tibialis posterior muscle is an important stabilizer of the internal arch of the foot and its dysfunction has been associated with musculoskeletal injuries of the foot [4]. The tibialis posterior has received attention because its relevance in equinovarus foot deformity in patients who had suffered a stroke. In fact, in patients with neurologic abnormalities, the tibialis posterior is one of the most frequently injected lower-extremity muscles for the management of spasticity [5].

Two approaches are commonly described in the literature for evaluating activity of the tibialis posterior muscle with an electromyographic needle: an anterior approach (crossing the interosseus membrane) and posterior/medial approach (crossing the flexor digitorum longus muscle) [6]. Each of these approaches has the potential risk of needling the anterior neurovascular (i.e., anterior tibial artery and veins and deep fibularis nerve) or posterior neurovascular (i.e., posterior tibial artery and veins) bundle, respectively. Different ultrasound studies determined the safety window and the depth of the tibialis posterior muscle with one [7, 8] or both [9, 10] approaches. All the studies concluded that the most favorable point for needle insertion into the tibialis posterior was the upper third of the leg for the anterior approach and a midpoint for the posterior/medial approach [7–10]. Table 1 shows safety window and depth of the tibialis posterior muscle reported by previous ultrasound studies.

**Table 1:** Safety window and depth to tibialis posterior in ultrasound-guided studies

Study	Anterior Approach (upper third point of the leg)		Posterior/Medial Approach (mid-point of the leg)	
	Safety window	Depth to tibialis posterior	Safety window	Depth to tibialis posterior
Won et al. [7]			1.47 ± 0.38 cm (0.62 cm to 2.16 cm)	2.31 ± 0.34 cm (1.57 cm to 3.16 cm)
Rha et al. [8]	1.09 ± 0.27 cm (0.64 cm to 2.13 cm)	3.34 ± 0.47 cm (2.47 cm to 4.66 cm)		
Rha et al. [9]	0.63 ± 0.12 cm (0.44 cm to 0.93 cm)	2.09 ± 0.17 cm (1.25 cm to 2.69 cm)	0.74 ± 0.23 cm (0.21 cm to 1.18 cm)	2.08 ± 0.38 cm (0.99 cm to 3.06 cm)
Won et al. [10]	1.29 ± 0.27 cm (0.7 cm to 2.12 cm)	2.61 ± 0.44 cm (0.79 cm to 3.56 cm)	1.45 ± 0.39 cm (0.31 cm to 2.78 cm)	2.43 ± 0.40 cm (1.58 cm to 3.52 cm)

These previous studies used electromyographic needles and an ultrasound guidance during the insertion [7–10]. It is important to note that ultrasound imaging is not available for daily clinical practice. Rather, most clinicians simply use a combination of superficial anatomical landmarks and knowledge of deeper anatomic structures to target the posterior tibialis and avoid the proximate neurovascular bundle. Three cadaver studies comparing the accuracy of needling the tibialis posterior muscle with/without ultrasound guiding reported that non-guided ultrasound needling accuracy is highly variable ranging from 39 to 50% [11–13]. These studies used electromyographic bevel needles and only used the posterior/medial approach for reaching the tibialis posterior muscle. No anatomical study has investigated if a solid needle, as clinically used by physical therapists during dry needling interventions, can accurately and safely reach the tibialis posterior muscle.

## Methods

### Objective

Our aims were to determine if a solid needle is able to accurately penetrate the tibialis posterior muscle using cadaver models; and to determine the safety of the needling procedure by calculating the distance between the needle and neurovascular bundles.

### Cadaveric sample

Eleven cryopreserved legs (45% females; mean age: 70, SD: 16 years) donated to an institutional university anatomy laboratory in Barcelona (Spain) were used. This study was approved by the Local Ethics Committee of Universitat Internacional de Catalunya (Cadaveric Barcelona Anatomy Lab CBA-2020–2). All experiments were conducted in accordance to guidelines of national/international/institutional or Declaration of Helsinki. To determine suitability for inclusion in the study, samples were visually checked for evidence of prior surgery, trauma, or any anatomical abnormalities by an experienced anatomist. Further, all cadavers were also required to be free of any disease or surgical procedure that would influence the integrity of the connective tissue in the lower extremity. The frozen samples were stored at -20ºC and were thawed at room temperature 24 h prior to the experiment. All cadaveric conservation procedures were supervised by the responsible of the anatomy laboratory.

### Needling procedure

**Fig. 1:**
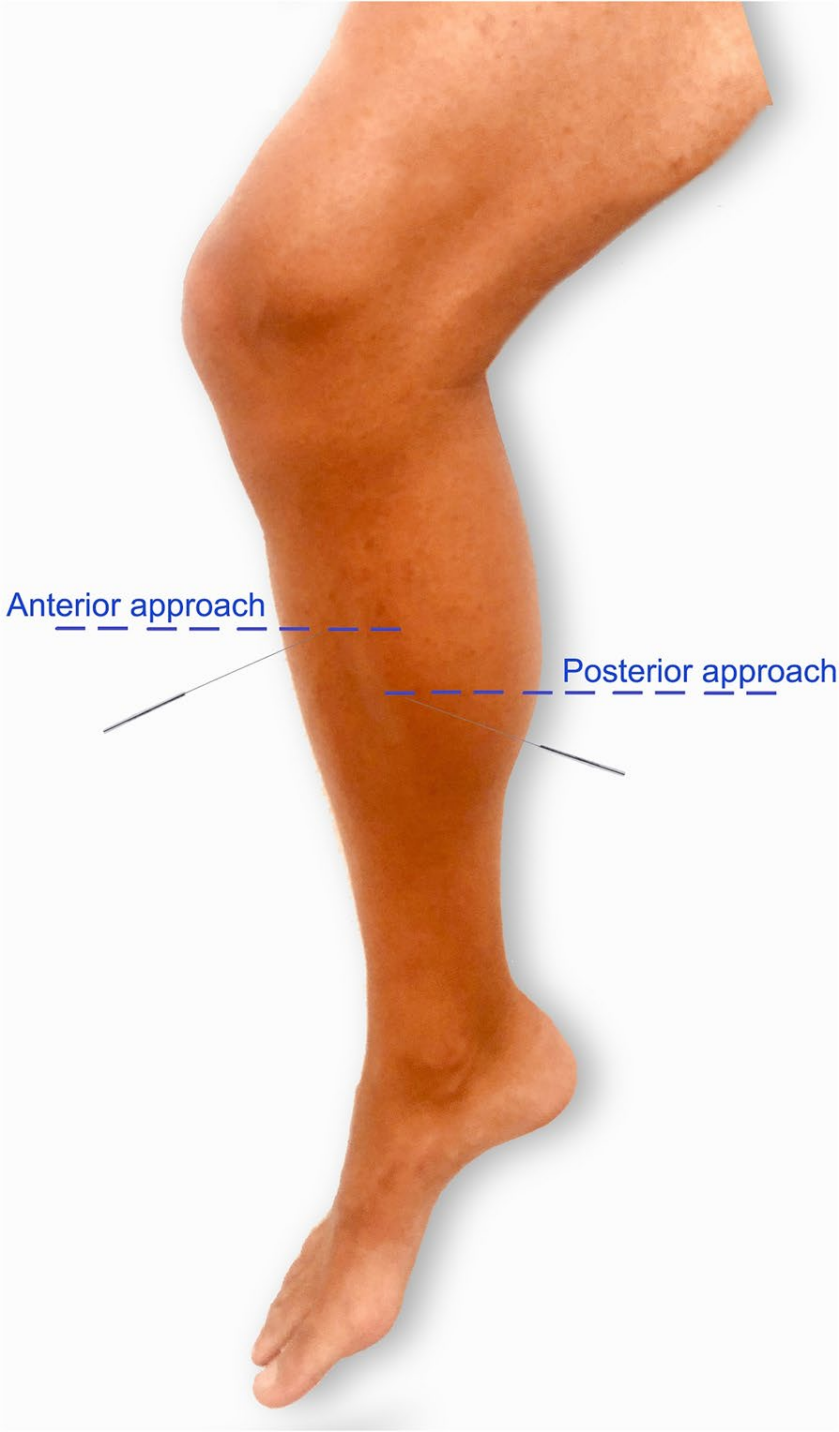
Point of needling insertion for the anterior and posterior/medial approach of the tibialis posterior muscle

Dry needling insertions were applied by a physical therapist with more than 10 years of experience with this intervention. Needling insertion targeting the tibialis posterior muscle was performed in the middle third of the leg for the posterior/medial approach and in the upper third of the leg for the anterior approach (Fig. 1) according to previous studies [7–10]. The length of the leg (from the tibial tubercle to the bimalleolar line) was first measured with a ruler.

**Fig. 2:**
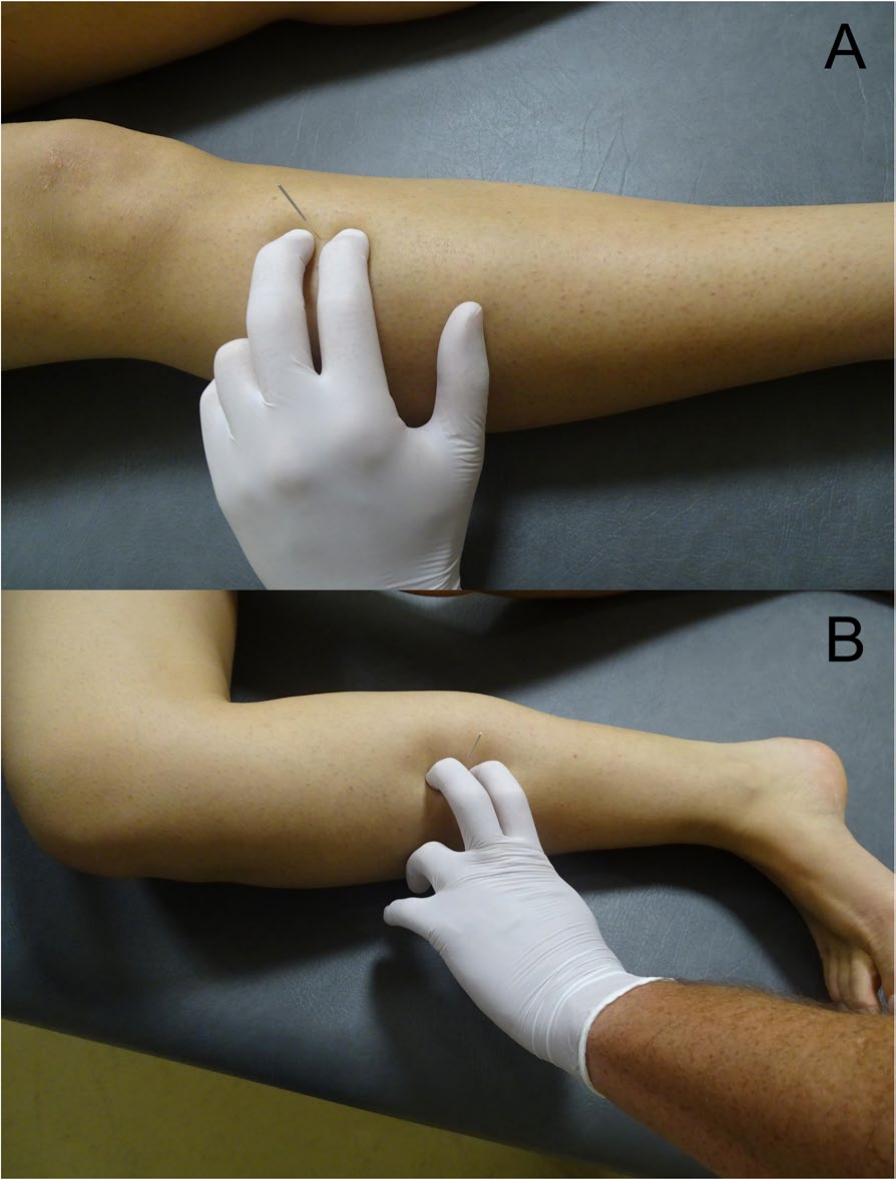
Application of the anterior **A** and posterior/medial **B** needling procedure of the tibialis posterior muscle

Sterile stainless-steel needles with a plastic cylindrical guide, 70 mm in length and 0.32 mm caliber were used for needling insertions. The length of the needle was arbitrary selected whereas the caliber is the regular one used for dry needling in clinical practice. For the anterior approach, the needle was inserted in the upper third of the leg close to the anterior-lateral aspect of the tibia and directed deeply in a posterior direction towards the tibialis posterior muscle crossing the interosseus membrane (Fig. 2A). For the posterior/ medial approach, the needle was inserted at the middle third of the leg close to the posterior aspect of the tibia and directed from a medial to lateral direction towards the tibialis posterior muscle crossing the flexor digitorum longus muscle (Fig. 2B). The needle was advanced to a depth judged clinically to be most likely in the tibialis posterior muscle for both approaches. Once the needle was inserted, latex was injected to mark where the tip of the needle was located to determine the accuracy of the insertion into the targeted muscle. In addition, the needle was left in situ to assess the path it followed to determine surrounding structures which were also penetrated by the needle before reaching the targeted muscle. For that purpose, an anatomical window of 4 cm x 4 cm was dissected in the targeted region once the needle and the injected latex were placed.

### Anatomical procedure

**Fig. 3:**
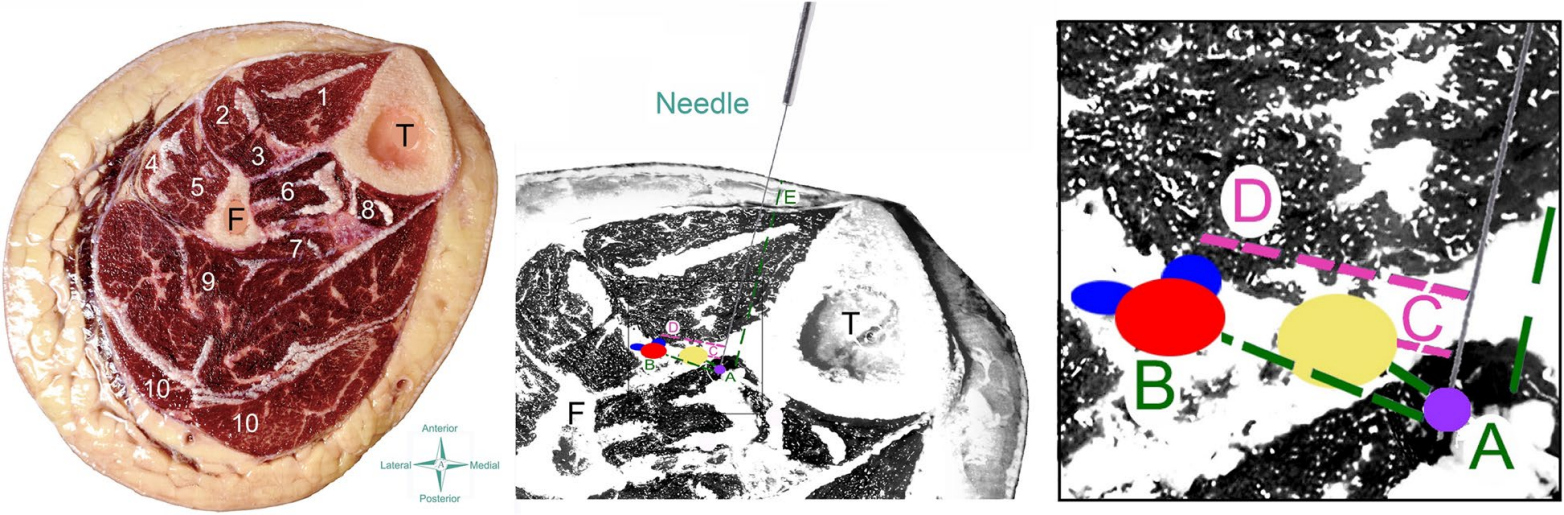
Scheme of the needling anterior approach on the cadaver (left) and anatomical draw (center and right). *T* Tibia, *F*: Fibula; 1: Tibialis anterior; 2: extensor digitorum longus; 3: extensor hallucis longus; 4: peroneus longus; 5: peroneus brevis; 6: tibialis posterior; 7: flexor hallucis longus; 8: flexor digitorum longus; 9: soleus; 10: gastrocnemius

**Fig. 4:**
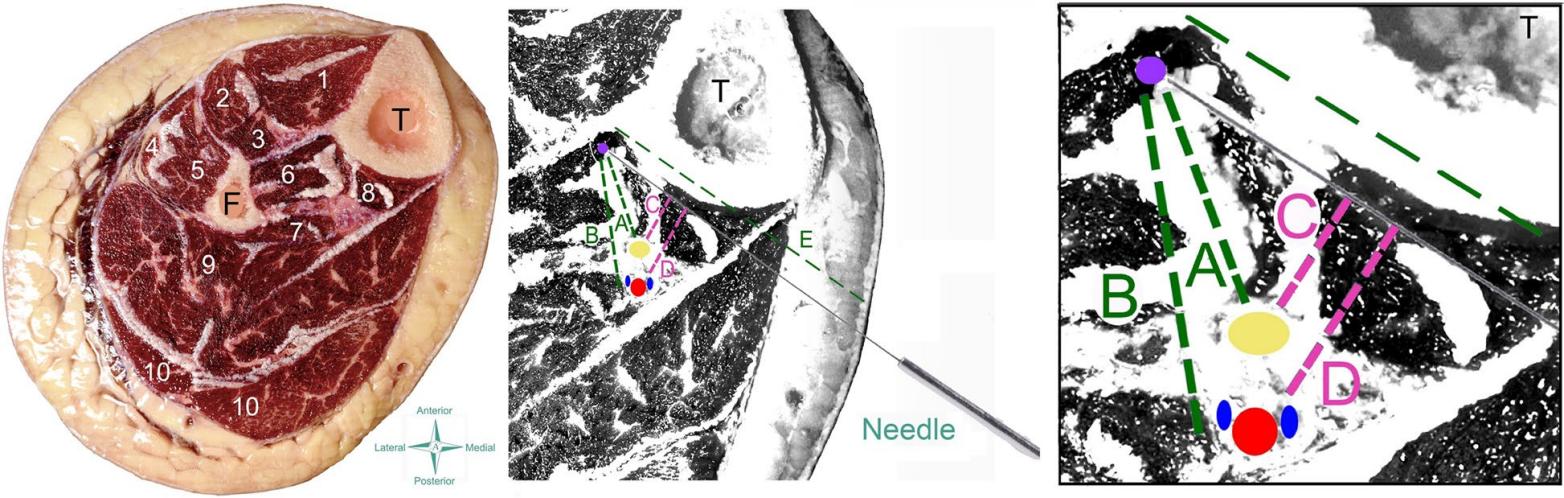
Scheme of the needling posterior/medial approach on the cadaver (left) and anatomical draw (center and right). T: Tibia; F: Fibula; 1: Tibialis anterior; 2: extensor digitorum longus; 3: extensor hallucis longus; 4: peroneus longus; 5: peroneus brevis; 6: tibialis posterior; 7: flexor hallucis longus; 8: flexor digitorum longus; 9: soleus; 10: gastrocnemius

The anatomical procedure used in the current cadaveric study were similar to that one previously used [14]. Cross-sectional anatomical dissections were photographed and after analyzed by photometry. The following measures in relation to the anterior (Fig. 3) or the posterior (Fig. 4) neurovascular bundle were calculated:

- 1) Needle tip to nerve distance (A): The distance (mm) between the tip of the needle and the tibial (posterior approach) or deep fibularis (anterior approach) nerve.
- 2) Needle tip to vascular bundle distance (B): The distance (mm) between the tip of the needle and the closest branch of the posterior or anterior tibial vascular bundle.
- 3) Needle path to nerve distance (C): The closest distance (mm) between the path of the needle and the tibial (posterior approach) or deep fibularis (anterior approach) nerve.
- 4) Needle path to vascular bundle distance (D): The closest distance (mm) between the path of the needle and the posterior or anterior tibial vascular bundle.

Additionally, the needle penetration (E, the length of the needle inserted to reach the tibialis posterior) was calculated.

### Statistical analysis

The statistical analysis was conducted with the SPSS statistical package (25.0 Version). Means, standard deviations, and 95% confidence intervals of the measurements were calculated. The comparison between the anterior and posterior/medial approaches were conducted with an analysis of covariance (ANCOVA) with age and gender as main covariates. The statistical significance was set at a value of *P* < 0.05.

## Results

**Fig. 5:**
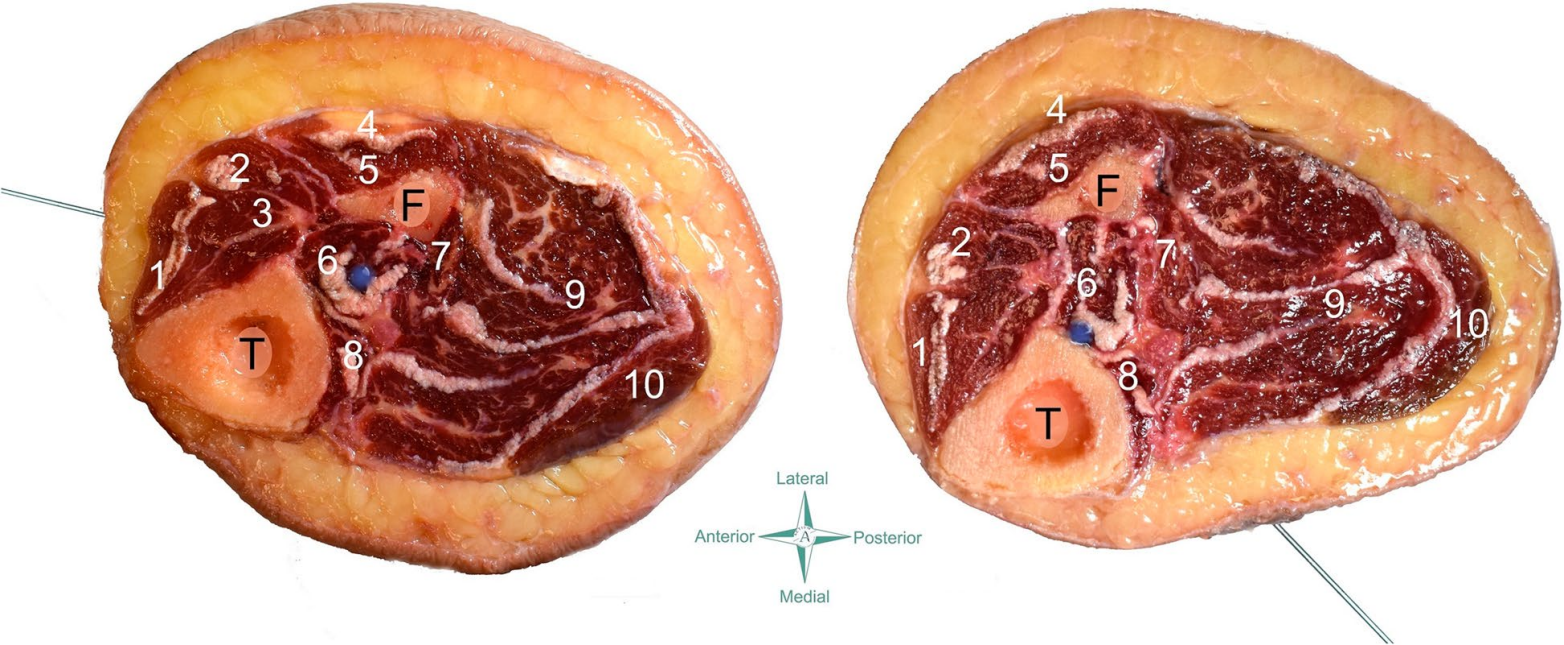
Anatomical study showing that the tip of the needle (blue latex points) targets the tibialis posterior with both anterior (left) and posterior/medial (right) approaches. T: Tibia; F: Fibula; 1: Tibialis anterior; 2: extensor digitorum longus; 3: extensor hallucis longus; 4: peroneus longus; 5: peroneus brevis; 6: tibialis posterior; 7: flexor hallucis longus; 8: flexor digitorum longus; 9: soleus; 10: gastrocnemius

From ten cadavers (twenty legs) donated to the anatomical laboratory from January to June 2021, needling of tibialis posterior muscle was conducted on eleven cryopreserved legs (5 females, 6 males; age: 70, SD: 16 years, 7 left and 4 right legs). The dissection revealed that the tip of the needle pierced the belly of the tibialis posterior muscle in all legs (accuracy of 100%) with both anterior (Fig. 5 left) and posterior/medial (Fig. 5 right) approaches. No neurovascular bundles were pierced in any of the eleven cadavers.

The distances from the needle to the neurovascular bundle are shown within Table 2. Distances to the neurovascular bundle were larger with the posterior/medial approach than with the anterior approach, reaching statistically significance for needle tip to nerve (mean difference:0.6 cm, 95%CI 0.35 to 0.85 cm, *F* = 22.394, *P* < 0.001) and vascular bundle (mean difference: 0.55 cm, 95%CI 0.3 to 0.8 cm, *F* = 16.580, *P* < 0.001) distances and needle path to vascular bundle distance (difference: 0.25 cm, 95%CI 0.1 to 0.4 cm, *F* = 5.204, *P* = 0.045). No significant differences in the depth of the tibialis posterior muscle were observed (*P* = 0.195). The inclusion of gender as covariate did not influence neither the distance for needle tip with nerve (*F* = 1.398, P = 0.252) and vascular bundle (*F* = 0.917, *P* = 0.351) nor the needle path to vascular bundle distance (*F* = 0.078, *P* = 0.783). Similarly, age did not influence the main results of the study (all, *P* > 0.124).

**Table 2:** Distances (mm) from the needle to the anterior or posterior neurovascular bundle assessed during dry needling approaches of the tibialis posterior muscle

**Anterior Approach (Upper Third of the Leg)**				
Depth to TP muscle (mm)	Needle Tip-DFN (cm)	Needle Tip-VB (cm)	Needle Path-DFN (cm)	Needle Path-VB (cm)
31.9 ± 6.2 (27.7 to 36.1 cm)	0.7 ± 0.3 (0.5 to 0.9 cm)	0.9 ± 0.35 (0.6 to 1.2 cm)	0.75 ± 0.3 (0.55 to 0.95 cm)	0.65 ± 0.3 (0.5 to 0.8 cm)
**Posterior/Medial Approach (Mid-Point of the Leg)**				
Depth to TP muscle (mm)	Needle Tip-TPN (cm)	Needle tip-VB (cm)	Needle Path-TPN (cm)	Needle Path-VB (cm)
35.1 ± 4.5 (32.0 to 38.2 cm)	1.35 ± 0.7 (1.15 to 1.55 cm)	1.45 ± 0.25 (1.3 to 1.6 cm)	1.0 ± 0.3 (0.8 to 1.2 cm)	0.95 ± 0.3 (0.75 to 1.15 cm)

*TP* Tibialis posterior, *DFN* Deep fibularis nerve, *VB* Vascular bundle, *TPN* Tibialis posterior nerve

## Discussion

The results of this cadaveric study found that the tip of the needle did pierce the tibialis posterior muscle during needle insertion with both a posterior/medial and anterior approach supporting the notion that this muscle can be accurately targeted with a needle during clinical application of dry needling. Our results agree with previous studies using an ultrasound-guide during the procedure and inserting electromyographic needle [7–10]. The main difference is that the use of ultrasound is not feasible for most clinicians in daily practice.

Current results are relevant since the tibialis posterior is not accessible to direct palpation due to its anatomical location; therefore, dry needling may represent the only therapeutic approach for this deep muscle. Although this study did not evaluate clinical effectiveness of the needling interventions, a recent meta-analysis found moderate-quality evidence supporting a positive effect of the application of dry needling on spasticity in the lower extremity in people who had suffered a stroke [15]. The tibialis posterior muscle is one muscle of the lower extremity that could receive dry needling. In fact, this muscle is the most commonly needled with botulinum toxin for the management of spasticity [5]. Future randomized controlled trials including dry needling of the tibialis posterior muscle are needed to determine the clinical effectiveness of these approaches.

Our results also support the safety of these needling procedures. We assessed the distances from the tip and the path of the needle to surrounding tissues potentially sensible to damage, such as nerves or vessels. Although no neurovascular bundles were pierced in any of our eleven cadavers, the posterior/medial approach resulted in a larger distance between the needle and neurovascular structures and, therefore, appears to be safer than the anterior approach. The results of our study agree with previous ultrasound imaging studies [7–10] that support the safety of performing dry needling tibialis posterior muscle. However, it should be considered that we assessed the distance from the needle to the neurovascular bundle, whereas the “safety window” in previous ultrasound studies was defined as the anatomical distance between the tibia and the neurovascular bundle. Therefore, it is expected that our distances would be smaller than the safety window. No previous study investigated the real distance between the needle and the neurovascular bundle. Based on current data, our study describes that clinical approaches of application of dry needling in the tibialis posterior [16], when applied by an experienced physical therapist, are accurate and safe. Clinicians should carefully control the angulation and orientation of the needle when targeting the tibialis posterior muscle since dry needling applications in our study were conducted close to the tibia bone [16]. Nevertheless, it is important to consider that this study did not specifically assess the safety of trigger point dry needling into the tibialis posterior muscle. In fact, we cannot say that dry needling anywhere in the tibialis posterior muscle is a safe procedure as we only investigated two distinct specific locations. Since trigger points can be located in any area of the muscle, it is conceivable that in other locations on the tibialis posterior muscle, the neurovascular bundles may be located in different locations relevant to superficial anatomy. However, it has been recently observed that the location of the needle (inside/outside of the trigger point) did not have a significant effect on spasticity and function of the upper extremity when dry needling shoulder muscles in individuals who had suffered a stroke [17]. We do not currently know if the same results would be found when needling the musculature of the lower extremity. Similarly, we should consider that if dry needling is applied on individuals who had suffered a stroke, the presence of spasticity-hypertonia could modify muscle tissue structure and, accordingly, some of the anatomical references considered in this study could change since cadavers have not exhibit spasticity.

Although this study supports accurate and safe placement of a solid needle on the tibialis posterior muscle, we should recognize some limitations. First, dissections were conducted in eleven single legs. Due to the small sample size, gender differences in needle placement were not evaluated. Second, although we used predetermined points of insertion based on previous studies [7–10], manual identification of landmarks is a requisite for a successful insertion into the targeted muscle. Third, all the needling insertions were conducted once by an experienced clinician and we do not know the accuracy or safety of this needling approach in the hands of unexperienced clinicians. 

## Conclusions

In conclusion, this cadaveric study suggests that needling of the tibialis posterior muscle can be accurately and safely conducted according to the guideline proposed. Safety seems to be larger with the posterior/medial approach when compared with the anterior approach. Clinicians should consider these results during their clinical practice.

## Data Availability

All data generated or analyzed during this study are included in this published article.

## References

[1] Gattie E, Cleland JA, Snodgrass S (2020). A survey of American physical therapists’ current practice of dry needling: Practice patterns and adverse events. Musculoskelet Sci Pract.

[2] Boyce D, Wempe H, Campbell C, Fuehne S, Zylstra E, Smith G (2020). Adverse events associated with therapeutic dry needling. Int J Sports Phys Ther.

[3] McManus R, Cleary M (2018). Radial nerve injury following dry needling. BMJ Case Rep.

[4] Kamiya T, Uchiyama E, Watanabe K, Suzuki D, Fujimiya M, Yamashita T (2012). Dynamic effect of the tibialis posterior muscle on the arch of the foot during cyclic axial loading. Clinical Biomechanic.

[5] Nalysnyk L, Papapetropoulos S, Rotella P, Simeone JC, Alter KE, Esquenazi A (2013). OnabotulinumtoxinA muscle injection patterns in adult spasticity: a systematic literature review. BMC Neurol.

[6] Lee HJ, Bach JR, DeLisa JA (1990). Needle electrode insertion into tibialis posterior: a new approach. Am J Phys Med Rehabil.

[7] Won SJ, Kim JY, Yoon JS (2011). Kim SJ Ultrasonographic evaluation of needle electromyography insertion into the tibialis posterior using a posterior approach. Arch Phys Med Rehabil.

[8] Rha DW, Im SH, Lee SC, Kim SK (2010). Needle insertion into the tibialis posterior: Ultrasonographic evaluation of an anterior approach. Arch Phys Med Rehabil.

[9] Rha DW, Park ES, Jung S, Lee SC, Suh M, Choi HS (2014). Comparison of ultrasound-guided anterior and posterior approaches for needle insertion into the tibialis posterior in hemiplegic children with spastic cerebral palsy. Am J Phys Med Rehabil.

[10] Won SJ, Yoon JS (2016). Approach for needle insertion into the tibialis posterior: An ultrasonography study. Muscle Nerve.

[11] Yun JS, Chung MJ, Kim HR, So JI, Park JE, Oh HM (2015). Accuracy of needle placement in cadavers: non-guided versus ultrasound-guided. Ann Rehabil Med.

[12] Boon AJ, Oney-Marlow TM, Murthy NS, Harper CM, McNamara TR, Smith J (2011). Accuracy of electromyography needle placement in cadavers: non-guided vs. ultrasound guided. Muscle Nerve.

[13] Haig AJ, Goodmurphy CW, Harris AR, Ruiz AP, Etemad J (2003). The accuracy of needle placement in lower-limb muscles: a blinded study. Arch Phys Med Rehabil.

[14] Rodríguez-Sanz J, Pérez-Bellmunt A, López-de-Celis C, Hidalgo-García C, Koppenhaver SL, Canet-Vintró M (2021). Accuracy and safety of dry needling placement in the popliteus muscle: A cadaveric study. Int J Clin Pract.

[15] Fernández-de-las-Peñas C, Pérez-Bellmunt A, Llurda-Almuzara L, Plaza-Manzano G, De-la-Llave-Rincón AI, Navarro-Santana MJ (2021). Is dry needling effective for the management of spasticity, pain, and motor function in post-stroke patients? A systematic review and meta-analysis. Pain Med.

[16] Dommerholt J, Fernandez-de-las-Peñas C (2019). Trigger point dry needling: an evidence and clinical-based approach.

[17] Hernández-Ortíz AR, Ponce-Luceño R, Sáez-Sánchez C, García-Sánchez O, Fernández-de-las-Peñas C (2020). de-la-Llave-Rincón AI, Changes in muscle tone, function, and pain in the chronic hemiparetic shoulder after dry needling within or outside trigger points in stroke patients: A crossover randomized clinical trial. Pain Med.

